# Predictive Significance of Serum MMP-9 in Papillary Thyroid Carcinoma

**DOI:** 10.1515/biol-2019-0031

**Published:** 2019-07-10

**Authors:** Dahai Xu, Chang Su, Liang Guo, He Yan, Shaokun Wang, Congwang Yuan, Guohui Chen, Li Pang, Nan Zhang

**Affiliations:** 1 Department of Emergency, The First Hospital of Jilin University, 71 Xinmin Street, Changchun, Jilin, 130021,China; 2 Department of Thyroid Surgery, the First Hospital of Jilin University, Changchun, Jilin, 130021, China; 3 Department of Pathology, the First Hospital of Jilin University, Changchun, Jilin, 130021, China; 4 Department of Pain, Yancheng First People’s Hospital, Yancheng, Jiangsu, 224000, China; 5 Department of Pathology, Jilin City People’s Hospital, Jilin, 132000, China

**Keywords:** matrix metalloproteinase-9, papillary thyroid carcinoma, disease-free survival, structural persistent/recurrent disease

## Abstract

**Objective:**

The incidence of papillary thyroid carcinoma (PTC) is increasing, and there are no reliable serum biomarkers for the diagnosis and prognosis of PTC. This study aimed to assess whether serum matrix metalloproteinase-9 (MMP-9) could serve as an auxiliary diagnostic/prognostic marker for PTC after total and partial thyroidectomy.

**Material and Methods:**

Postoperative serum MMP-9 concentrations were measured in 182 male patients with PTC, 86 male patients with benign thyroid nodule (BTN), and 62 male healthy controls (HCs). Multivariate logistic regression and Cox regression were applied to evaluate the correlation between variables. The performance of serum MMP-9 in diagnosing PTC and predicting structural persistent/recurrent disease (SPRD) during 48 months of follow-up after initial surgery was evaluated by receiving operating characteristic curve analysis.

**Results:**

The median serum MMP-9 concentration in the PTC group (79.45 ng/ml) was significantly higher than those in the BTN group (47.35 ng/ml) and HC group (47.71 ng/ml). The area under the curve (AUC) for predicting PTC from BTN was 0.852 at a cut-off value of 60.59 ng/ml. Serum MMP-9 was negatively correlated with disease-free survival (OR 1.026, P=0.001). Serum MMP-9 exhibited good performance in predicting SPRD at a cutoff value of 99.25 ng/ml with an AUC of 0.818. Advanced TNM stage (OR 31.371, P=0.019) and serum MMP-9 ≥99.25 ng/ml (OR 4.103, P=0.022) were independent risk factors for SPRD.

**Conclusions:**

Serum MMP-9 potentially represents a good predictive biomarker for PTC diagnosis and prognosis after thyroidectomy in Chinese male patients for whom radio-imaging indicates suspected PTC.

## 1 Introduction

Papillary thyroid carcinoma (PTC) accounts for the majority (80‒85%) of thyroid cancers [1]. Despite a fair prognosis in most cases, PTC can differentiate into some aggressive and lethal thyroid carcinomas, such as poorly differentiated thyroid carcinoma or anaplastic (undifferentiated) thyroid carcinoma [2]. In addition, a high recurrence rate of 30% and cancer-related mortality rate of 8% have been reported for PTC [3]. Although several biomarkers, such as micro-RNA451 [4], metallothioneins, Notch1 [5] and HBME-1, have been reported for diagnosis of PTC in recent years, their efficacy has yet to be validated. At present, there is no standard biomarker for diagnosing PTC and predicting the prognosis of PTC in clinical practice. Therefore, it is necessary to find a reliable diagnostic and prognostic molecular marker for PTC.

Matrix metalloproteinase-9 (MMP-9, 92-kDa gelatinase/type IV collagenase) is involved in the degradation and remodeling of extracellular matrix, which has been verified to be associated with carcinogenesis [6, 7, 8, 9]. Over-expression of MMP-9 in serum and/or tissue has been found in oral squamous cell carcinoma and cervical cancer [6, 10]. In addition, the tissue MMP-9 level is a prognostic factor for poor clinical outcomes in patients with various carcinomas, including hepatic breast cancer metastases, prostate cancer, salivary adenoid cystic carcinoma, and gastric cancer [7, 11, 12, 13], because its level is typically associated with recurrence, advanced clinical stage, presence of invasion or metastasis, and shorter survival time. On the contrary, inhibition of MMP-9 leads to attenuated tumor cell growth and decreased invasive and migratory abilities of cancer cells [14, 15, 16].

Although specific findings have been inconsistent, several studies have proposed MMP-9 as a predictor of thyroid cancer [17, 18, 19]. MMP-9 expression in thyroid tissue was found to be associated with aggressive features and prognosis, including lymph node metastasis, tumor status, TNM stage and degree of tumor infiltration, likely via the ROCK/MMP-9 pathway [17, 20, 21]. Consistent with the tissue MMP-9 level, the serum MMP-9 level was reportedly increased in patients with PTC, especially in those with lymph node involvement [22, 23]. Another study confirmed the association between a high serum MMP-9 level and poorer prognosis in PTC patients after radiofrequency ablation [24]. However, the validity of preoperative circulating MMP-9 in predicting the prognosis of PTC after total or partial thyroidectomy has not been established.

Based on the previous studies, we speculated that preoperative circulating MMP-9 levels may be helpful in predicting the prognosis of PTC. In this study, we measured circulating MMP-9 levels in patients with PTC and assessed the diagnostic accuracy and prognostic value of serum MMP-9 for PTC.

## 2 Materials and methods

### 2.1 Study population

From July 2012 and July 2013, male patients who were newly diagnosed with primary PTC at the First Hospital of Jilin University were consecutively enrolled in this study. Consecutive patients with BTN and healthy individuals without thyroid nodules were recruited as controls. Radiological methods, including ultrasound, computed tomography (CT), and magnetic resonance imaging, were implemented for the diagnosis of PTC and BTN. The final diagnosis was confirmed by histopathological findings from fine needle aspiration biopsy and/or thyroid tissue after total or partial thyroidectomy. Patients were excluded if they had the following conditions: a) other primary carcinomas; b) secondary PTC via metastasis from another carcinoma; c) radiation therapy before admission; d) severe organ dysfunction, such as renal failure, heart failure; or e) incomplete data.

**Informed consent**: Informed consent has been obtained from all individuals included in this study.

**Ethical approval**: The research related to human use has been complied with all the relevant national regulations, institutional policies and in accordance the tenets of the Helsinki Declaration, and has been approved by the ethics committee of the First Hospital of Jilin University.

The clinical TNM stage of PTC was graded according to the 7^th^ Edition of the American Joint Committee on Cancer (AJCC) staging system [25]. Early-stage PTC was defined as TNM stage I/II, and advanced-stage as stage III/IV. For PTC patients who underwent total thyroidectomy, bilateral central-compartment neck dissection was performed. Lateral or modified neck dissection was done in patients with cervical lymph node metastasis based on definitive clinical and/or imaging evidence. All surgical procedures were performed by the same team of surgeons.

Postoperative evaluations were routinely conducted every 3 months lasting 4 years. The follow-up was conducted from July 2012 to July 2017 after initial surgery. PTC recurrence was monitored by neck ultrasound and lung CT/chest X-ray. Serum thyroglobulin (Tg) and thyroglobulin antibody (TgAb) levels were routinely measured every 3 months. The modified criteria for evaluating disease status were as follows [26]: 1) no evidence of disease: Tg <1 ng/ml without evidence of structural disease; 2) indeterminate response: detectable but low TgAb with absence of structural disease; 3) biochemically persistent disease: Tg ≥1 ng/ml but without evidence of structural disease; 4) structurally persistent disease: metastasis (locoregional or distant) irrespective of Tg levels; 5) recurrence events: structural evidence of disease that was identified after a period of no evidence of disease. Disease-free survival was defined as being alive without evidence of recurrence or metastatic disease at the end of the follow-up period. Survival time without structural persistent/recurrent disease (SPRD) was defined as the interval between the initial treatment date and structurally persistent disease, recurrent disease, or last follow-up.

### 2.2 Blood sampling and determination of serum MMP-9 concentration

Preoperative fasting venous blood samples were collected from patients with PTC or BTN at the time of diagnosis. Samples were allowed to sit for 30 min to allow proper clot formation and then subjected to centrifugation at 3000 × g for 10 minutes. The sera were frozen and stored at −70°C until analysis.

MMP-9 concentration was measured in triplicate using a commercially available enzyme-linked immunoassay kit (Human Quantikine MMP-9 Immunoassay, R&D Systems Inc., Minneapolis, MN, USA) according to the manufacturer’s instructions. Anti-human MMP-9 antibodies can bind both the inactive pro-form (92 kDa) and active form (82 kDa) of MMP-9. The limit of detection for the serum MMP-9 level was 0.156 ng/ml. The intra-assay coefficient of variation (CV) was <5%, and the inter-assay CV was 6‒7% in this study.

### 2.3 Statistical analysis

SPSS 18.0 (SPSS, Inc., Chicago, IL, USA) and GraphPad Prism 5.01 software (GraphPad, Inc., San Diego, CA) were used for statistical analyses. Continuous data are presented as mean ± standard deviation (SD) if normally distributed; non-normally distributed data are presented as median and interquartile range (IQR). The categorical data are expressed as frequencies and percentages. For normally distributed data, independent t test or analysis of variance (ANOVA) was used, and for data not normally distributed, nonparametric test was applied. Proportions were compared using the chi-squared test or nonparametric test.

Correlations between serum MMP-9 concentration and clinicopathological characteristics were analyzed by Pearson correlation test. The diagnostic performance of serum MMP-9 in the prediction of PTC was evaluated in terms of sensitivity, specificity, positive predictive value (PPV), and negative predictive value (NPV) based on a receiver operating characteristic (ROC) curve. The optimal cutoff value of serum MMP-9 for diagnosis was determined according to the Youden index [27]. Survival curves were plotted using the Kaplan–Meier method and compared using the log-rank test. Multivariate Cox proportional hazard analysis was performed to identify risk factors for SPRD, with results reported as odds ratios (ORs) and 95% confidence intervals (CIs). All statistical tests were two-sided, and P<0.05 was considered statistically significant.

## 3 Results

### 3.1 Patient characteristics

This study included 182 patients with PTC, 86 patients with BTN, and 62 healthy controls (HCs). The clinicopathological characteristics of the participants are summarized in **Table 1**.

**Table 1:** Clinicopathological characteristics of the study subjects, n (%)

Variables	PTC	BTN	HC
	(n = 182)	(n = 86)	(n = 62)
Age			
<45 years	111 (61.0)	15 (17.4)	34 (54.8)
≥45 years	71 (39.0)	71 (82.6)	28 (45.2)
Tumor size			
≤1 cm	110 (60.4)		
>1 cm	72 (39.6)		
Capsule invasion			
No	61 (33.5)		
Yes	121 (66.5)		
Multifocality			
Unifocal	84 (46.2)		
Multifocal	98 (53.8)		
Nodal status			
N0	72 (39.6)		
CLNM	102 (56.0)		
LLNM	47 (25.8)		
Extrathyroidal invasion			
Negative	144 (79.2)		
Microscopic	29 (15.9)		
Macroscopic	9 (4.9)		
Vascular invasion			
No	166 (91.2)		
Yes	16 (8.8)		
Distant metastasis			
No	172 (94.5)		
Yes	10 (5.5)		
TNM stage			
I+II	141 (77.5)		
III+IV	41 (22.5)		

PTC: papillary thyroid carcinoma; BTN: benign thyroid nodule; HC: healthy control; TNM, tumor-node-metastasis; CLNM: central lymph node metastasis; LLNM: lateral lymph node metastasis.

### 3.2 Serum MMP-9 concentrations in different groups

**Figure 1:**
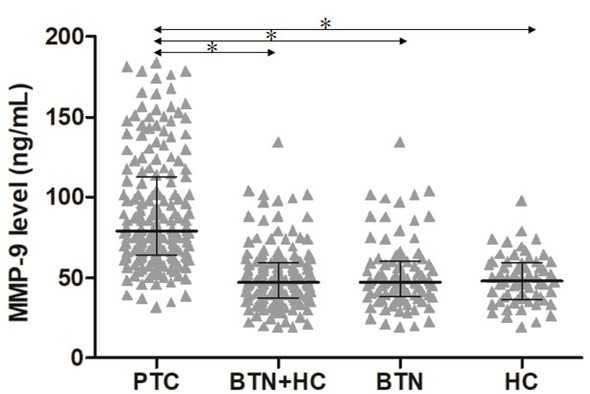
Serum MMP-9 concentrations in PTC, BTN and/or HC groups. PTC, papillary thyroid carcinoma; BTN, benign thyroid nodule; HC, healthy control. The black horizontal lines represent the median value of measurements, and the error bars represent interquartile range. * p<0.05.

The median serum MMP-9 concentration in the PTC group was 79.45 ng/ml (IQR, 64.06‒113.15 ng/ml, which was significantly higher than those of the BTN group (47.35, 38.05‒68.14 ng/ml; P<0.001), HC group (47.71, 36.70‒59.52 ng/ml; P<0.001), and BTN+HC group (47.54, 37.42‒59.67 ng/ml; P<0.001), as shown in **Figure 1**. No statistical difference in serum MMP-9 concentration was found between the BTN and HC groups (P≥0.05).

### 3.3 Correlations between serum MMP-9 concentration and clinicopathological features

Pearson correlation analysis demonstrated that the serum MMP-9 concentration was positively correlated with malignancy (P<0.001), tumor size (P=0.029), lateral lymph node metastasis (P<0.001), extrathyroidal invasion (P=0.022), presence of distant metastasis (P=0.022), and TNM stage (III+IV) (P<0.001), as shown in **Table 2**.

**Table 2:** Correlation between serum MMP-9 level and clinicopathological characteristics by Pearson correlation test

Variables	Pearson correlation	P value
Malignancy (PTC *vs*. HC+BTN)	0.563	**<0.001**
Tumor size (≥1 cm *vs*.<1 cm)	0.162	**0.029**
Capsule invasion (yes *vs*. no)	0.126	0.091
Multifocality (yes *vs*. no)	0.025	0.737
Central lymph node metastasis (yes *vs*. no)	0.002	0.978
Lateral lymph node metastasis (yes *vs*. no)	0.285	**<0.001**
Extrathyroidal invasion (yes *vs*. no)	0.169	**0.022**
Vascular invasion (yes *vs*. no)	0.099	0.185
Distant metastasis (yes *vs*. no)	0.218	**0.003**
TNM stage (III+IV *vs*. I+II)	0.415	**<0.001**

PTC: papillary thyroid carcinoma; BTN: benign thyroid nodule; HC: healthy control; TNM, tumor-node-metastasis.

### 3.4 Performance of serum MMP-9 in the diagnosis of PTC

**Figure 2:**
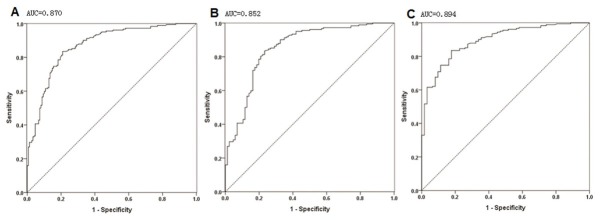
ROC curves for the diagnosis of PTC based on serum MMP-9 concentration. The diagnostic performance of serum MMP-9 in differentiating PTC from BTN+HC (A), BTN (B), and HC (C). ROC, receiver operating characteristic; PTC, papillary thyroid carcinoma; BTN, benign thyroid nodule; HC, healthy control.

ROC curves were constructed to judge the diagnostic performance of serum MMP-9 in differentiating PTC from BTN and BTN+HC (**Figure 2**). The AUC value for predicting PTC from BTN was 0.852 (95% CI 0.800‒0.904) with an optimum cut-off serum MMP-9 value of 60.59 ng/ml. Similarly, the AUC values were 0.870 (95% CI 0.831‒0.908) and 0.894 (95% CI 0.852‒0.937) for differential diagnosis of PTC from HC and BTN+HC, respectively, with an optimum cut-off value of serum MMP-9 of 60.65 ng/ml (**Table 3**).

**Table 3:** Diagnostic performance of serum MMP-9 in differentiating PTC from BTN and/or HC

Group	Optimum cutoff value	AUC	Sensitivity	Specificity	PPV	NPV	LR+	LR-
	(ng/ml)	(95% CI)	(%)	(%)	(%)	(%)		
PTC *vs*. BTN+HC	60.65	0.870 (0.831-0.908)	83.5	79.1	83.1	79.6	3.995	0.209
PTC *vs*. BTN	60.59	0.852 (0.800-0.904)	83.5	76.7	88.4	68.8	3.584	0.215
PTC *vs*. HC	60.65	0.894 (0.852-0.937)	83.5	82.3	93.3	63.0	4.718	0.200

PTC: papillary thyroid carcinoma; BTN: benign thyroid nodule; HC: healthy control; AUC: area under curve; PPV: positive predictive value; NPV: negative predictive value; LR+: positive likelihood ratio; LR-: negative likelihood ratio.

### 3.5 Clinicopathological features of patients with PTC stratified by serum MMP-9 concentration

The PTC patients were stratified into low MMP-9 (<60.59 ng/ml) and high MMP-9 (≥60.59 ng/ml) subsets based on the optimum cut-off value of serum MMP-9. PTC patients with a high serum MMP-9 level were more likely to have a large tumor size (>1 cm), lateral lymph node metastasis, extrathyroidal invasion, and advanced TNM stage, as shown in **Table 4**.

**Table 4:** Clinicopathological characteristics of PTC patients stratified by serum MMP-9 level at a cut-off of 60.59 ng/ml, n (%)

	Serum MMP-9 (ng/ml)		

Variables	Low level <60.59	High level ≥60.59	P value
	(n=30)	(n=152)	
Age			0.129
<45 years	22 (73.3)	89 (58.6)	
≥45 years	8 (26.7)	63 (41.4)	
Tumor size (cm)			**0.047**
≤1 cm	23 (76.7)	87 (57.2)	
>1 cm	7 (23.3)	65 (42.8)	
Capsule invasion			0.410
No	12 (40.0)	49 (32.2)	
Yes	18 (60.0)	103 (67.8)	
Multifocality			0.459
No	12 (40.0)	72 (47.4)	
Yes	18 (60.0)	80 (52.6)	
Central lymph node metastasis			0.743
No	14 (46.7)	66 (43.4)	
Yes	16 (53.3)	86 (56.6)	
Lateral lymph node metastasis			**0.030**
No	27 (90.0)	108 (71.1)	
Yes	3 (10.0)	44 (28.9)	
Extrathyroidal invasion			**0.036**
No	28 (93.3)	116 (76.3)	
Yes	2 (6.7)	36 (23.7)	
Vascular invasion			0.923
No	28 (93.3)	138 (90.8)	
Yes	2 (6.7)	14 (9.2)	
Distant metastasis			0.314
No	30 (100.0)	142 (93.4)	
Yes	0 (0.00)	10 (6.6)	
TNM stage			**0.037**
I+II	29 (96.7)	112 (73.7)	
III+IV	1 (3.3)	40 (26.3)	

PTC: papillary thyroid carcinoma; BTN: benign thyroid nodule; HC: healthy control; TNM, tumor-node-metastasis. P values were determined by Chi-square test.

**Figure 3:**
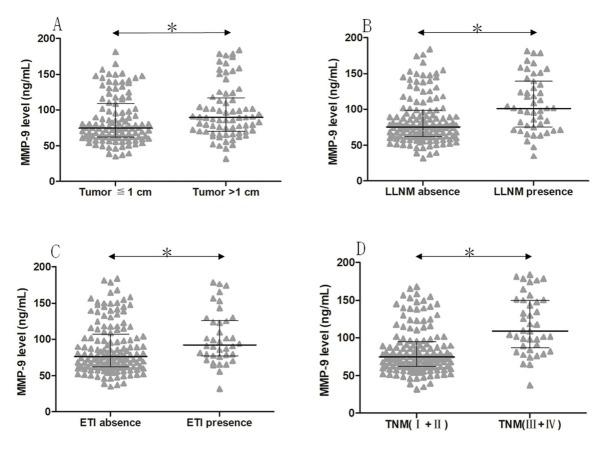
Serum MMP-9 levels. Comparison of serum MMP-9 level was made between patients with tumor size > 1 cm vs. ≤ 1cm (A), presence vs. absence of LLNM (B), presence vs. absence of ETI (C) or early TNM stage (I+II) vs. advanced TNM stage (III+IV) (D). LLNM, lateral lymph node metastasis; ETI, extrathyroidal invasion. The black horizontal lines indicate the median values of measurements, and the error bars indicate interquartile range. * p<0.05.

On the other hand, patients with tumor size >1 cm had a higher serum MMP-9 level than those tumor size ≤1 cm (median 89.34 ng/ml, IQR 69.98‒116.99 vs. median 74.91 ng/ml, IQR 61.91‒109.03; P=0.013). Similarly, patients with lateral lymph node metastasis (median 101.46 ng/ml, IQR 75.41‒139.36 vs. median 75.08, IQR 62.30‒98.27), extrathyroidal invasion (92.50 ng/ml, IQR 76.95-126.11 vs. median 76.28 ng/ml, IQR 62.35‒107.40), or advanced TNM stage (median 109.37 ng/ml, IQR 89.24-149.74 vs. median 74.56 ng/ml, IQR 64.22‒94.80) had a higher serum MMP-9 level than those patients without (all *P*<0.05), as shown in **Figure 3**.

### 3.6 Association between serum MMP-9 and disease-free survival

The patients were followed up for 4 years. The multivariate logistic regression model revealed that serum MMP-9 (OR 1.026, 95% CI 1.011‒1.042, P=0.001), capsule invasion (OR 2.987, 95% CI 1.058‒8.433, P=0.039), CLNM (OR 5.089, 95% CI 1.716‒15.091, P=0.003), and vascular invasion (OR 13.623, 95% CI 1.745‒106.334, P=0.013) were negatively correlated with disease-free survival (**Table 5**).

**Table 5:** Correlation between clinicopathological characteristics and disease-free survival by multivariate logistic regression

Variables	OR	95% CI	P value
Age	1.047	0.990-1.106	0.107
BMI	0.955	0.837-1.090	0.499
MMP-9	1.026	1.011-1.042	**0.001**
Tumor size (>1 cm *vs*. ≤1 cm)	1.184	0.399-3.512	0.760
Capsule invasion (yes *vs*. no)	2.987	1.058-8.433	**0.039**
Multifocality (yes *vs*. no)	0.683	0.276-1.690	0.409
Central lymph node metastasis (yes *vs*. no)	5.089	1.716-15.091	**0.003**
Lateral lymph node metastasis (yes *vs*. no)	1.043	0.379-2.873	0.935
Extrathyroidal invasion (yes *vs*. no)	1.576	0.478-5.194	0.454
Vascular invasion (yes *vs*. no)	13.623	1.745-106.334	**0.013**
Distant metastasis (yes *vs*. no)	4.262	0.564-32.15	0.160
TNM stage (III+IV *vs*. I+II)	1.823	0.507-6.553	0.357

BMI: body mass index; TNM, tumor-node-metastasis. Age, BMI, and MMP-9 concentrations are continuous data.

### 3.7 Serum MMP-9 in the prediction of SPRD

**Figure 4:**
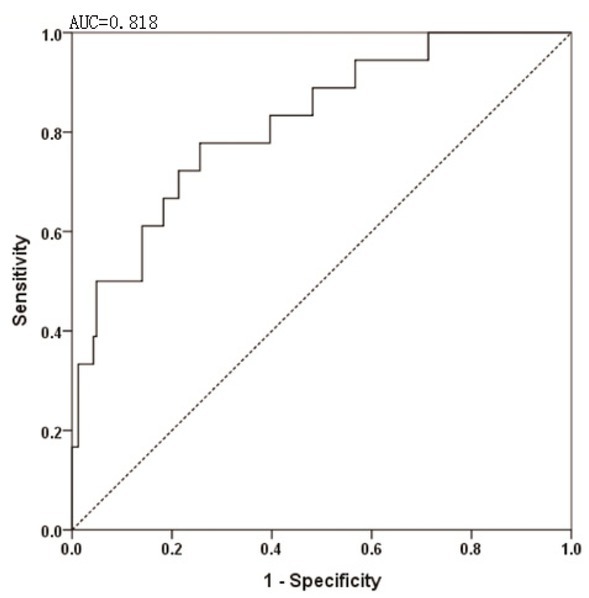
ROC curve for serum MMP-9 concentration in predicting SPRD during 4-year follow-up. Serum MMP-9 exhibited good performance in predicting SPRD at a cut-off value of 99.25 ng/ml with an AUC of 0.818 (95% CI 0.715‒0.922), a sensitivity of 77.8%, and specificity of 73.8%. SPRD, structural persistent/recurrent disease.

ROC curve analysis was performed to examine the accuracy of the serum MMP-9 level for predicting SPRD (**Figure 2**). Serum MMP-9 exhibited good performance in predicting SPRD at a cutoff value of 99.25 ng/mL with an AUC of 0.818 (95% CI 0.715‒0.922), a sensitivity of 77.8%, and specificity of 73.8%, as shown in **Figure 4**.

**Figure 5:**
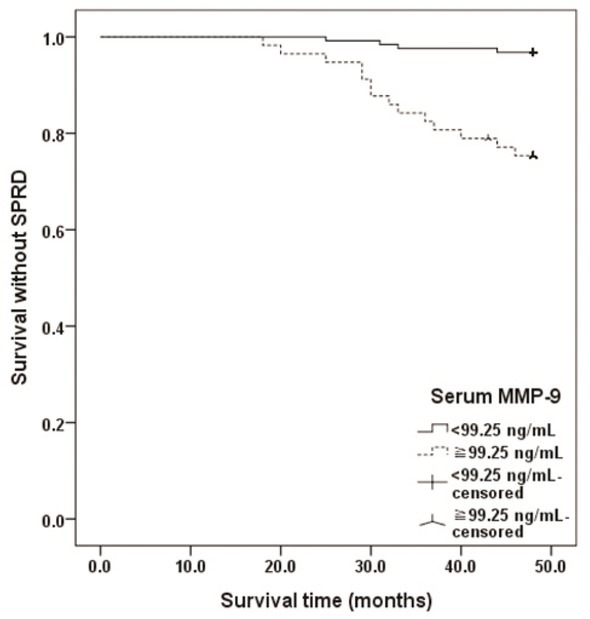
Survival analysis for no SPRD in PTC patients by a cut-off value of serum MMP-9 (<99.25 ng/ml *vs*. ≥99.25 ng/ml).

Compared with patients with a lower serum MMP-9 level (<99.25 ng/ml), patients with a high serum MMP-9 level (≥99.25 ng/ml) were found to have a shorter survival without SPRD (47.53±2.88 months *vs*. 44.00±7.94 months; **Figure 5**).

Patients were more likely to develop SPRD if they were older (age ≥45 years; P=0.02) or had a larger tumor size (>1 cm, P=0.03), capsule invasion (P=0.08), central lymph node metastasis (P=0.014), extrathyroidal invasion (P<0.001), vascular invasion (P<0.001), distant metastasis (P=0.28), and advanced TNM stage (P<0.001; **Table 6**).

**Table 6:** Clinicopathological characteristics of patients with or without SPRD, n (%)

Variables	No SPRD	SPRD	P value
Age			**0.002**
<45 years	106 (64.6)	5 (27.8)	
≥45 years	58 (35.4)	13 (72.2)	
Tumor size			**0.003**
≤1 cm	105 (64.0)	5 (27.8)	
>1 cm	59 (36.0)	13 (72.2)	
Capsule invasion			**0.008**
No	60 (36.6)	1 (5.6)	
Yes	104 (63.4)	17 (94.4)	
Multifocality			0.250
No	78 (47.6)	6 (33.3)	
Yes	86 (52.4)	12 (66.7)	
Central lymph node metastasis			**0.014**
No	77 (47.0)	3 (16.7)	
Yes	87 (53.0)	15 (83.3)	
Lateral lymph node metastasis			0.629
No	123 (75.0)	12 (66.7)	
Yes	41 (25.0)	6 (33.3)	
Extrathyroidal invasion			**<0.001**
Negative	136 (82.9)	8 (44.4)	
Microscopic	25 (15.2)	4 (22.2)	
Macroscopic	3 (1.8)	6 (33.3)	
Vascular invasion			**<0.001**
No	155 (94.5)	11 (61.1)	
Yes	9 (5.5)	7 (38.9)	
Distant metastasis			**0.028**
No	157 (95.7)	15 (83.3)	
Yes	7 (4.3)	3 (16.7)	
TNM stage			**<0.001**
I+II	137 (83.5)	4 (22.2)	
III+IV	27 (16.5)	14 (77.8)	

SPRD: structural persistent/recurrent disease; TNM, tumor-node-metastasis. P values were determined by Chi-square.

### 3.8 Risk factors for predicting SPRD by Cox proportional hazards regression analysis

The univariate Cox proportional hazard regression model showed that the following factors were significantly correlated with SPRD: age (P=0.005), tumor size (P=0.007), capsule invasion (P=0.031), central lymph node metastasis (P=0.026), extrathyroidal invasion (P=0.001), vascular invasion (P<0.001), advanced TNM stage (P<0.001), and serum MMP-9 level (P<0.001). Next, these factors were included in a multivariate Cox regression model. Only advanced TNM stage (OR 31.371, 95 CI 1.746‒563.611, P=0.019) and serum MMP-9 ≥99.25 ng/ml (OR 4.103, 95% CI 1.225‒13.740, P=0.022) were independent risk factors for predicting SPRD (**Table 7**).

**Table 7:** Cox proportional hazards regression analysis for SPRD in patients with PTC

	Univariate analysis		Multivariate analysis	

	OR (95% CI)	P value	OR (95% CI)	P value
Age (≥45 *vs*. <45 years)	4.366 (1.556-12.250)	**0.005**	0.166 (0.010-2.739)	0.209
BMI	1.049 (0.915-1.203)	0.493		
(≥25 *vs*. <25 kg/m^2^)				
Tumor size	4.155 (1.481-11.656)	**0.007**	1.325 (0.340-5.166)	0.686
(>1 *vs*. ≤1cm)				
Capsule invasion (yes *vs*. no)	9.229 (1.228-69.359)	**0.031**	4.666 (0.589-36.950)	0.145
Multifocality (yes *vs*. no)	1.741 (0.653-4.639)	0.267		
CLNM (yes *vs*. no)	4.081 (1.181-14.098)	**0.026**	2.133 (0.449-10.133)	0.341
LLNM (yes *vs*. no)	1.474 (0.553-3.928)	0.438		
Extrathyroidal invasion (yes *vs*. no)	5.070 (2.000-12.854)	**0.001**	1.407 (0.394-5.023)	0.599
Vascular invasion (yes *vs*. no)	7.232 (2.801-18.676)	**0.000**	2.551 (0.747-8.713)	0.135
Distant metastasis (yes *vs*. no)	4.081 (1.179-14.122)	**0.026**	1.248 (0.241-6.465)	0.792
TNM stage (III+IV *vs*. I+II)	14.480 (4.760-44.050)	**0.000**	31.371 (1.746-563.611)	**0.019**
Serum MMP-9 (≥99.25 *vs*. <99.25 ng/ml)	8.630 (2.839-26.233)	**0.000**	4.103 (1.225-13.740)	**0.022**

PTC: papillary thyroid carcinoma; SPRD: structural persistent/recurrent disease; CLNM: central lymph node metastasis; LLNM: lateral lymph node metastasis; TNM, tumor-node-metastasis.

## 4 Discussion

In this study, we found that an increased preoperative serum MMP-9 level might help in the differential diagnosis of PTC and prediction of SPRD at cut-off values of 60.59 ng/ml and 99.25 ng/ml, respectively, in Chinese male patients with PTC. Also, a high preoperative serum MMP-9 level was an independent risk factor for SPRD. Currently, molecular detection within tumor tissues is highly restricted in daily clinical work because of its invasiveness and unavailability. In contrast, blood-borne biomarkers are convenient, cost-efficient, and widely acceptable [28]. Thus, with good sensitivity and specificity according to ROC curve analysis, serum MMP-9 measurement represents a potential auxiliary diagnostic method for PTC that may be helpful for evaluating the risk of development and outcomes of PTC in patients with suspected thyroid nodules by palpation or imaging evidence, especially when histopathological examination is temporarily unavailable.

MMP-9 or gelatinase B is a classical enzyme that belongs to the zinc-metalloproteinase family, and it participates in the degradation of the extracellular matrix [29]. Since malignant progression of tumor is typically accompanied by degradation of extracellular matrix,

MMP-9 is believed to be involved in the development of diverse tumor types [30, 31, 32]. Up-regulation of tissue MMP-9 has been demonstrated in PTC by immunohistochemical analysis [33]. Consistently, we have found that the serum MMP-9 level is significantly higher in patients with PTC than in those with BTN [19, 22]. Similar results can be found in head and neck squamous cell carcinoma [34]. Overexpression of MMP-9 in different sample types has revealed the common feature of MMP-9 across a variety of tumor types, probably due to secretion of MMP-9 into body fluids. Despite the convenience and easy availability of blood sampling, the sensitivity and specificity of serum MMP-9 versus tissue MMP-9 for PTC diagnosis should be further verified. In a previous study, immunohistochemistry showed positive staining for active MMP-9 in 57% of PTC samples [17]. Another study revealed that positive immunostaining for MMP-9 was observed in 92.4% (61/66) of PTC tissue samples versus 20% (8/40) of BTN tissue samples [33]. Our study also revealed the good diagnostic performance of serum MMP-9 with a sensitivity of 83.5% and specificity of 76.7% at the cut-off value of 60.59 ng/ml. However, it is impossible at present to discern whether tissue or serum MMP-9 achieves better accuracy for PTC diagnosis due to different sample sizes and populations among studies.

Given that MMP-9 can degrade components of the basement membrane, it is considered to be involved in processes requiring basement membrane disruption, such as tumor invasion and tissue infiltration of T lymphocytes [23, 35]. Our findings indicated that serum MMP-9 is positively associated with tumor size, lymph node metastasis, extrathyroidal invasion, and TNM stage. These results are consistent with the previous findings, which suggested a positive association between positive immunostaining for MMP-9 and older age (≥45 years), advanced clinical stage (III–IV), and larger tumor diameter (≥2 cm) in PTC [33]. The results above indicated that MMP-9 is a potential indicator of unfavorable PTC progression.

Elevated expression of MMP-9 has been linked to shortened survival in several types of cancers. Immunohistochemical results have exhibited that overexpression of MMP-9 correlates with poor outcome as evaluated by disease-free survival in colorectal carcinoma [36]. A meta-analysis showed that MMP-9 overexpression is significantly associated with poor overall survival (HR: 1.65) and shortened disease-free survival (HR: 1.61) in nasopharyngeal carcinoma [37]. In this study, we found that a higher preoperative serum MMP-9 level was associated with worse disease-free survival and was an independent risk factor for SPRD. Our findings are consistent with others in primary hepatic carcinoma [38].

Although MMP-9 has been implicated in carcinogenesis, the molecular mechanisms remain largely unclear. The following molecular mechanisms may be involved in the carcinogenesis of PTC: First, as we mentioned before, MMP-9 is involved in the degradation of type IV collagen and denatured collagen (critical component of basement membrane), which contributes to the aggressiveness of carcinoma [7, 29, 30]. Secondly, neovascularization, including tumor angiogenesis, is an essential step for the metastasis of thyroid carcinoma [39]. Angiogenesis initiates the transition from a preneoplastic stage to a neoplastic stage [40]. MMP-9 is involved in angiogenesis, as it promotes the availability of vascular endothelial cell growth factor (VEGF) in malignant tumors, which boosts tumor progression [29]. In addition, VEGF enhances the secretion of MMP-9 from stromal cells and provides necessary nutrients for neovascularization [38]. Thirdly, MMP-9 promotes tumor progression by cleaving a variety of substrates, including growth factor precursors, growth factor binding proteins, receptor tyrosine kinases, cell adhesion molecules, and other proteinases [17]. Finally, some epigenetic changes, such as decreased promoter methylation of MMP-9, are found to be associated with carcinogenesis [32]. Further studies are needed to explore its mechanism.

This study has several strengths. First, to the best of our knowledge, we for the first time demonstrated the performance of serum MMP-9 in PTC prognosis after total and partial thyroidectomy. Second, only consecutive male subjects were enrolled in this study, which avoided gender-based bias. However, our study also has some limitations. First, this study was conducted in a single-center with a relatively small sample size and without enrollment of female participants. Second, this study explored the associations between the serum MMP-9 concentration and clinicopathological features of PTC patients as well as their clinical outcomes. However, some unmeasured factors that could have possibly influenced the observed association cannot be completely eliminated. For instance, the possible heterogeneity in postoperative treatment among patients was not taken into consideration when correlations between MMP-9 level and prognosis were analyzed. In addition, no control with a known biomarker was set due to the lack of a standard serum marker for PTC as yet.

In conclusion, this study provided preliminary evidence that the preoperative serum MMP-9 concentration may be a potentially useful biomarker for both diagnosing PTC and predicting its prognosis in Chinese male patients. However, the potential application of MMP-9 in clinical practice needs further validation. More in-depth and randomized studies with a large sample size are required to confirm and supplement these findings.
